# Ultrasensitive Detection of PSA Using Antibodies in Crowding Polyelectrolyte Multilayers on a Silicon Nanowire Field-Effect Transistor

**DOI:** 10.3390/polym16030332

**Published:** 2024-01-25

**Authors:** Galina V. Presnova, Denis E. Presnov, Mariya M. Ulyashova, Ilia I. Tsiniaikin, Artem S. Trifonov, Ekaterina V. Skorb, Vladimir A. Krupenin, Oleg V. Snigirev, Maya Yu. Rubtsova

**Affiliations:** 1 Department of Chemistry, Lomonosov Moscow State University, 119991 Moscow, Russia; gkovba@enzyme.chem.msu.ru (G.V.P.); mmu@enzyme.chem.msu.ru (M.M.U.); 2 Faculty of Physics, Lomonosov Moscow State University, 119991 Moscow, Russia; ii.tcinyaykin@physics.msu.ru (I.I.T.); artem.trifonov@physics.msu.ru (A.S.T.); krupenin@phys.msu.ru (V.A.K.); snigirev.oleg@physics.msu.ru (O.V.S.); 3 D.V. Skobeltsyn Institute of Nuclear Physics, Lomonosov Moscow State University, 119991 Moscow, Russia; 4 Infochemistry Scientific Center of ITMO University, 191002 Saint Petersburg, Russia; skorb@itmo.ru

**Keywords:** polyelecrolytes, macromolecular crowding, antibodies, silicon nanowire field-effect transistor, prostate-specific antigen (PSA), immunosensor

## Abstract

Immunosensors based on field-effect transistors with nanowire channels (NWFETs) provide fast and real-time detection of a variety of biomarkers without the need for additional labels. The key feature of the developed immunosensor is the coating of silicon NWs with multilayers of polyelectrolytes (polyethylenimine (PEI) and polystyrene sulfonate (PSS)). By causing a macromolecular crowding effect, it ensures the “soft fixation” of the antibodies into the 3-D matrix of the oppositely charged layers. We investigated the interaction of prostate-specific antigen (PSA), a biomarker of prostate cancer, and antibodies adsorbed in the PEI and PSS matrix. In order to visualize the formation of immune complexes between polyelectrolyte layers using SEM and AFM techniques, we employed a second clone of antibodies labeled with gold nanoparticles. PSA was able to penetrate the matrix and concentrate close to the surface layer, which is crucial for its detection on the nanowires. Additionally, this provides the optimal orientation of the antibodies’ active centers for interacting with the antigen and improves their mobility. NWFETs were fabricated from SOI material using high-resolution e-beam lithography, thin film vacuum deposition, and reactive-ion etching processes. The immunosensor was characterized by a high sensitivity to pH (71 mV/pH) and an ultra-low limit of detection (LOD) of 0.04 fg/mL for PSA. The response of the immunosensor takes less than a minute, and the measurement is carried out in real time. This approach seems promising for further investigation of its applicability for early screening of prostate cancer and POC systems.

## 1. Introduction

The development of personalized medicine and modern healthcare necessitates the creation of novel techniques for real-time detection of low concentrations of particular biomarkers (proteins, nucleic acids, etc.) in clinical biochemistry to facilitate early diagnosis. The development of biosensors based on field-effect transistors with a nanowire channel (NWFETs) has advanced significantly in the last few years. The key feature is the shape of the channel—a very thin semiconductor nanowire; which allows the detection of charged biomolecules on or near its surface by changing the nanowire conductivity. The thinner the nanowire, the larger part of its volume the field penetrates, significantly changing its conductivity, which leads to an increase in the transistor sensitivity. Distinct electronic characteristics, nanoscale size, and real-time measurement without biomarker labeling make NWFETs an appealing diagnostic platform [1]. Numerous studies have demonstrated silicon nanowire advantages over other sensor types since the first reported experiments [2]. They are unique in that they have high charge/field sensitivity [3], the ability to measure in a wide temperature range up to room temperature, which is crucial for studying biological objects, and spatial resolution limited only by the diameter of the nanowire [4,5,6]. Biosensor platforms are continually improving as a result of ongoing advancements in nanotechnology techniques. The primary benefits of silicon-on-insulator (SOI) nanowires are their reproducibility and reliability in the manufacturing process, as well as their compatibility with industrial silicon technology, which enables the production of measuring electronics in close proximity to the sensor. Numerous FET-based devices have been created recently for use in biomedical diagnostics, with the aim of identifying different biomarkers of cancer, acute myocardial infarction, bacterial and viral infections, and others [7,8,9,10,11].

In order to identify biological molecules, NWFETs frequently use antibodies to capture particular biomarkers, and the formation of immune complexes changes the nanowires’ conductivity. The sensitivity of immunosensors based on NWFETs is very high; they can detect individual molecules at a level ranging from micromoles to attomoles or even lower up to detecting single molecules [12,13,14,15].

Optimizing the response of these biosensors, increasing the signal-to-noise ratio, and overcoming the theoretical Nernst limit receive particular attention [16,17,18,19]. However, there is a significant restriction concerning the Debye screening length (λ_D_), which is the average distance at which the electric field enters the buffer. The λ_D_ characterizes the thickness of an electrical double layer close to the gate dielectric/buffer interface in an electrolyte buffer [20,21]. The ionic strength of the buffer solution has a significant impact on this parameter [22]. For instance, in high-ionic strength solutions such as human serum (analog 1 × PBS), λ_D_ is approximately 0.7 nm, which prevents the efficient use of complete immunoglobulin molecules, which have a thickness of approximately 2 nm [23]. This results in the necessity of using low-ionic-strength buffers (λ_D_ in 0.01 PBS is approximately 7 nm). However, under these circumstances, the effectiveness of antibody interactions with the analyte may be reduced.

By covering the planar nanowires with additional layers with gaps and nanopores, it is possible to reduce the shielding effect of the charged molecules being analyzed [24]. Applying polymers, like polyethylene glycol, to cover the surface of nanowires is an additional tactic that has been demonstrated to extend the effective Debye screening length [25]. There might be more benefits to coating biosensor surfaces with polymers. Antibodies that are immobilized or adsorb directly onto the solid surface of the immunosensor are known to exhibit decreased affinity for the antigen as a result of steric effects and decreased active center mobility, particularly when interacting with large biomarker molecules. In order to improve antibody binding capacity, the surface of the sensor can be modified with charged polyelectrolytes adsorbing proteins through electrostatic interactions [26,27]. Antibodies can be “soft fixed” in a 3D matrix on the surface of sensors formed by multilayers of oppositely charged polyelectrolytes, preserving some degree of “internal disorder” and the polypeptide chains’ mobility [28]. Because of the phenomenon known as “molecular crowding”, the high specificity and activity of antibodies placed in layers of charged polyelectrolytes are ensured [29]. Due to the small volume of the hydrogel nanolayer as well as the “tightness” effect, the interaction between molecules in such an environment occurs more quickly than in the volume of the solution. In the process of developing an electrochemical biosensor for the detection of the encephalitis virus, the benefits of immobilizing specific antibodies into the matrix of successive layers of positively charged polyethylenimine (PEI) and negatively charged polystyrene sulfonate (PSS) were recently demonstrated [30].

This work investigates the coating of silicon with layers of PEI and PSS polyelectrolytes to create NWFETs for detecting prostate-specific antigen (PSA). PSA represents a molecular biomarker for the clinical diagnosis of prostate cancer. PSA serum concentrations higher than the upper limit of 4.0 ng/mL, particularly if they exceed 10 ng/mL, suggest a possible cancer risk and need to be confirmed further. Furthermore, a postoperative serum PSA level exceeding 0.1 ng/mL would indicate a recurrence risk for cancer, making it especially crucial to reduce the detection limit for PSA to nanogram levels or below.

## 2. Materials and Methods

### 2.1. Materials

All chemicals and organic solvents were of analytical grade. Poly(sodium 4-styrenesulfonate) (PSS, Mw 500 kDa), polyethyleneimine (PEI, Mw 70 kDa, 50 wt% water solution), tetrachloroauric acid, bovine serum albumin (BSA), and Tween-20 were purchased from Sigma-Aldrich (St. Louis, MO, USA). Sodium citrate dihydrate was purchased from MP Biomedicals (Eschwege, Germany). PSA and two clones of mouse monoclonal antibodies to PSA and monoclonal antibodies to TSH (nonspecific to PSA) were provided by JSC “NVO Immunotek” (Moscow, Russia). The specificity of the antibodies towards PSA was confirmed by the manufacturer. All water used in experiments was purified with a Milli-Q system (Millipore, Billerica, MA, USA).

Gold nanoparticles (AuNPs) with a diameter of 20–25 nm were prepared by the Frens method [31], based on the reduction of chloroauric acid by sodium citrate. They were characterized spectrophotometrically (λ_max_ = 521 nm and λ_max_ = 519 nm) and by using scanning electron microscopy (SEM).

Conjugates of AuNPs with antibody half-fragments containing thiol groups were prepared as described in [8].

### 2.2. Silicon Surface Modification by Polyelectrolytes and Adsorption of Antibodies in the Matrix of Polymers

The concentrations of the reagents were used in accordance with the method developed earlier for the formation of a polyelectrolyte matrix of PEI and PSS on the surface of screen-printed electrodes [30]. A total of 5 μL of PEI solution (1 mg/mL in water) was spotted onto the silicon surface, dried, and rinsed three times with water to remove excess PEI. Then, 5 μL of antibodies against PSA (100 μg/mL) in PBS pH 7.4 was dropped on the modified surface of silicon, dried, and washed three times with PBS containing 0.1% Tween20 (PBST). In the next step, 5 μL of PSS solution (2 mg/mL in water) was added, dried, and washed similarly to PEI solution.

### 2.3. Characterization of a Silicon Surface Covered by Multilayers of Polyelectrolytes and Antibodies

The characterization of silicon samples coated with layers of polyelectrolytes with adsorbed antibodies was performed using successive stages of sandwich immunoassay with the use of AuNPs as labels by SEM and AFM methods. A piece of silicon covered with layers of polyelectrolytes and antibodies adsorbed between them was incubated in a solution of PSA (20 ng/mL in PBST) at 37 °C for 1 h. After washing, it was incubated in a solution of a conjugate of second antibodies with AuNPs at 37 °C for 1 h. Antibodies nonspecific to PSA and their conjugate with AuNPS were used as controls.

SEM images of the silicon surface were obtained using a field emission scanning electron microscopy (FESEM) Supra40 (Carl Zeiss, Germany) with an InLens secondary electron detector integrated in the microscope column.

The tapping mode of SmartSPM-1000 (AIST-NT, Novato, CA, USA) was used for obtaining the topography profiles. The diamond single crystal cantilevers AFM Probe ART™ D300 used in this study have a typical spring constant of 10 N/m and a rounded tip of 5–10 nm radius. The spring constant of each cantilever was determined using a technique based on measuring the change in resonant frequency of the fundamental mode of vibration [32]. The scratching was studied by performing several (40–80) scratching runs at a load of 1.0 µN. The scratch depth was estimated as the maximum level decreasing in the relatively unscratched area of the film.

### 2.4. Fabrication of NW FETs and Electrical Measurements

For the fabrication of our nanowire field-effect transistor, we used a well-known top-down approach [33,34] that involved electron-beam lithography to create all components of the structure, reactive ion etching to transfer the pattern into the silicon layer, and thin film deposition. The process is fully CMOS-compatible; see the Supplementary Materials for more details. Brief description: commercially available SoiTec Unibond^®^ silicon-on-insulator (SOI) wafers had a 110 nm-thick top Si layer with a resistivity of 10 Ω cm. It was isolated from the 725 μm thick Si handle wafer with the same resistivity by a 200 nm thick buried oxide layer of SiO_2_. This wafer was used as a bottom gate to adjust the working point of the transistor. The transistor structures were formed in the top active monocrystalline silicon layer by electron-beam lithography using FESEM Supra40 (Carl Zeiss) equipped with the ELPHY Quantum (RAITH) pattern generator. A nanowire pattern 80–90 nm wide and 3–5 μm long was drawn in a thin layer of positive electron beam resist A4 PMMA 950K. After development, a 15 nm Al film was deposited in open windows, and the rest was removed by a lift-off process. This metal layer was used as a mask during the following anisotropic reactive ion etching of the active Si layer in fluorine-containing plasma and later dissolved in a weak alkaline solution. The top views of the nanowire transistor structure were obtained using FESEM. Particular attention was paid to insulating the titanium conductive leads from the gate handle substrate and the reaction liquid medium. To do this, additional layers of silicon oxide were successively deposited onto the sample (excluding its central part). For measurements, the sample was fixed on a special holder, and the contacts were connected by ultrasonic bonding. The aluminum wires were then completely coated with sealant.

Modification of NWs with polyelectrolytes and antibodies was performed as described in Section 2.2. Measurements of the transistor transport characteristics, as well as measurements of the NWFET responses based on them, were carried out using a home-made measuring system consisting of a preamplifier, a voltage setting unit, and an interface unit providing communication with the control computer. Measurements were performed at positive voltages on the main (substrate) gate, which corresponds to the inverse (electronic) conductivity channel of the transistor. The AgCl reference electrode served as an additional gate for the transistor; it was immersed in the solution drop to adjust its operating point.

The operating point on the signal characteristic of the transistor was determined by the setting voltages (V_sd_, V_g_, and V_ref_), and the current changes caused by external influence (changes in pH, formation of immune complexes) were recorded by a current preamplifier. The preamplifier’s own low noise level (30 fA/√Hz) made it possible to register ultra-low current changes up to picoamps. Details are presented in the Supplementary Materials.

### 2.5. Determination of PSA by the NWFETs

Measurements on the FETs with nanowire channels modified by polyelectrolytes were performed in static mode without fluid flow, which provides stability and reproducible real-time response. First, a baseline was obtained from the flowing buffer solution (0.01 × PBS buffer pH 7.4). After it was stabilized, various concentrations of PSA in the same buffer were applied, and the change in the current was registered. BSA (1 ng/mL) was used for control experiments. The conductivity of the NW was modulated by the voltages at the main gate silicon substrate of the SOI wafer and the AgCl reference electrode. The limit of PSA detection (LOD) was calculated as the mean conductivity for the PSA-free solution (0 ng/mL PSA) plus 3 SD (*n* = 10).

## 3. Results and Discussion

### 3.1. Principle of Immunosensor Based on Field-Effect Transistors with Nanowire Channel Coated by Layers of Oppositely Charged Polyelectrolytes

The basis for determining the analyte on NW FETs is its direct capture by immobilized antibodies on a nanowire surface. Total charge on the NW surface varies as a result of immune complexes formation, which leads to a change in NW conductivity and variations in transport current. Measurements on nanowires were carried out in solutions with low ionic strength because the Debye length was strongly dependent on it. We propose to coat the surface of nanowires with charged polyelectrolytes to facilitate the functioning of antibodies. Antibody molecules can be “fixed softly” in a pseudo-homogeneous medium consisting of layers of oppositely charged polyelectrolytes, which preserves their mobility more than immobilization via covalent bonds.

**Figure 1:**
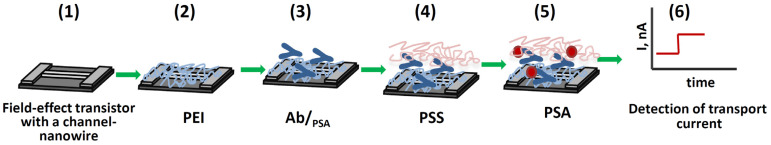
A scheme for determining PSA on a field-effect transistor with a nanowire using multilayers of charged polyelectrolytes. (**1**) Fabrication of a field-effect transistor with a nanowire channel; (**2**) The surface of the silicon nanowire is covered with positively charged polyelectrolyte PEI; (**3**) Antibodies to the analyte are adsorbed; (**4**) The surface is covered with negatively charged polyelectrolyte PSS; (**5**) PSA solution is passed through the nanowire; (**6**) A change in the transport current of the nanowire is registered.

The principle of determining PSA on immunosensors based on FETs with NW channels modified by layers of charged polyelectrolytes is shown in Figure 1. Modification of silicon with polyelectrolytes was carried out using a well-known layer-by-layer technique. Cationic polyelectrolyte PEI was suggested for the formation of the first layer on the surface, as its advantage in terms of uniform distribution onto semiconductor surfaces with thicknesses of microns was shown earlier [35]. IgG molecules have a negative charge at pH 7.4 [36], which is favorable for their interaction with PEI. Since the groups of negatively charged PSS completely dissociate in an experimental setting and help to form stable protein-polyelectrolyte complexes [37,38], it was used as a second polyelectrolyte. We examined the simplest matrix, which contained one layer of PEI and one layer of PSS, in order to identify the thinnest modifying layer of the surface relevant to the desired Debye length.

The formation of a matrix of charged polyelectrolytes and antibodies involves several stages of spotting reagent solutions (PEI, PSA-specific antibodies, and PSS) on a silicon surface, drying, and several washings. As a result, we obtained a polyelectrolyte matrix in which the recognizing molecules of the immunosersor are located between the layers of oppositely charged polyelectrolytes.

Immune complexes are formed when the PSA solution is passed through a transistor with a nanowire channel. These complexes alter the conductivity of the transistor in response to changes in the electric charge on or near the nanowire’s surface.

We examined the thickness of the polyelectrolyte layers and the formation of immune complexes when passing specific and non-specific proteins in order to investigate the preservation of antibody specificity and the reproducibility of the procedure.

### 3.2. Modification of Silicon Surface by Antibodies Adsorbed in a Matrix of Charged Polyelectrolytes and Study of the Formation of Immune Complexes

**Figure 2:**
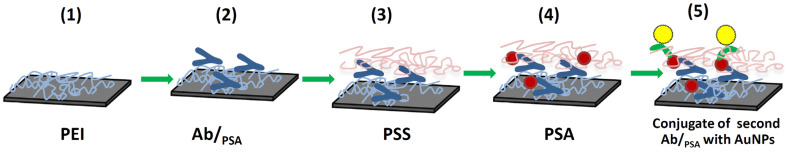
The scheme of silicon modification by the layers of polyelectrolytes and antibodies and the study of immune complex formation. (**1**) The surface of the silicon is covered with PEI; (**2**) Antibodies to PSA are adsorbed; (**3**) The surface is covered with PSS; (**4**) Incubation with PSA solution and washing; (**5**) Incubation with a conjugate of second antibodies labeled with gold nanoparticles and washing.

At the first stage, silicon samples (small pieces of silicon wafer) were utilized to study the polymer matrix’s composition. The silicon surface was first coated with a layer of PEI, then covered with antibodies to PSA, and finally covered with a layer of PSS. In order to estimate the binding density of antibodies adsorbed in the matrix, we investigated the formation of sandwich immune complexes, composed of an antigen molecule and two molecules of antibodies, one immobilized on the modified silicon and the other labeled with AuNPs of 25 nm. Figure 2 illustrates the scheme for studying immune complex formation. To obtain sandwich immune complexes, modified silicon samples were successively incubated in a PSA solution and then in a solution of the second antibody to PSA with AuNPs. The conjugate of the second antibody was produced through covalent binding of AuNPs with their half-fragments containing thiol groups.

The thickness of the formed layers on the silicon surface as well as its morphology were investigated using SEM (a piece of silicon was split lengthwise and analyzed at an angle of at least 70°) and AFM (scanning of a silicon surface before and after making a scratch by applying 1 µN constant force along a 1 µm line). The combination of the two methods allowed us to determine the following parameters: the thickness of the PEI layer, the thickness of the matrix, the distribution of AuNPs on the surface, and their location inside the matrix.

The values of the matrix thickness on the silicon surface and the surface roughness are presented in Table 1. Native silicon is always covered with a thin layer of its oxide, which is characterized by a very small roughness of 0.5 nm. We changed the concentration of PEI to obtain a layer of minimal thickness (Figure S1). The thin layer of PEI that formed on the silicon surface had a thickness of 6 nm and was composed of half silicon oxide and half polymer. The optimal concentration of PEI for this process was 1 mg/mL (Figure S1). The surface of the layer has become notably rougher, but it has remained smooth overall. The slightly larger values of layer thicknesses obtained in AFM are apparently explained by the fact that AFM measured the depth of the scratch in unstressed layers, but SEM measured a slightly stressed (as a result of a splitting) edge of layers.

With good convergence of the two methods, the thickness of a matrix of two polyelectrolytes with adsorbed specific antibodies was increased up to about 22 nm, while the roughness remained relatively unchanged.

After interaction with the antigen, the layer’s surface became more granular, and its thickness somewhat increased. A notable increase in the layer thickness value’s standard deviation should be noted. This may be a sign of immune complexes formation in the matrix layer and is most likely the result of sufficiently large antibody and antigen molecules.

There was no discernible increase in layer thickness following interaction with the conjugate of the second antibody labeled with AuNPs (without accounting for nanoparticle size). The height of the matrix layer has also become more consistent at the same time. The determination of the PEI and the matrix thickness performed for different sample preparations (Figure S2) showed good reproducibility of the method.

Control experiments showed that the layer thickness was less when using non-specific antibodies and their conjugates compared to the use of specific antibodies. This suggests that formation of immune complexes results in a slight increase in matrix layer thickness and a decrease in density.

The layer height was obtained less in both control experiments using non-specific antibodies to PSA at the stage of polyelectrolyte matrix formation and as second antibodies than when using antibodies specific to PSA. At the same time, the layer thickness was lower compared to the formation of triple sandwich complexes from one PSA molecule and two antibody molecules. This is most likely due to the formation of fewer double complexes from one antigen molecule and one antibody molecule in control experiments compared to the number of triple sandwich complexes.

**Figure 3:**
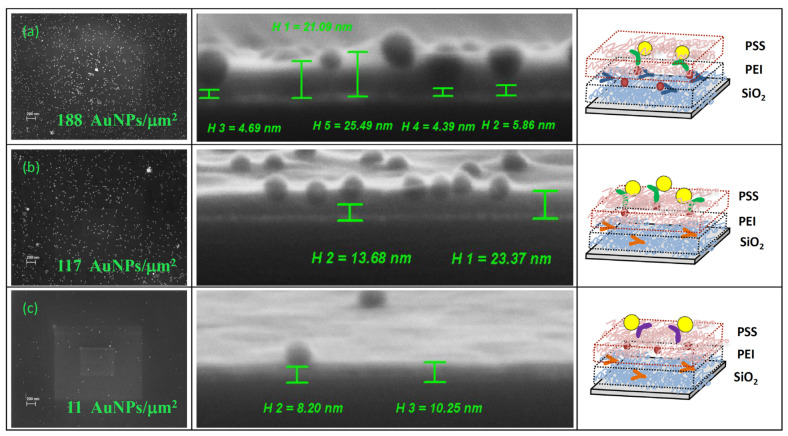
SEM images of silicon samples modified with polyelectrolytes with immune complexes of various compositions. (**a**) antibodies to PSA were adsorbed in the polyelectrolyte matrix, then a silicon sample was incubated in a solution of PSA and a conjugate of antibodies specific to PSA with AuNPs; (**b**) non-specific antibodies were adsorbed in the matrix, then a silicon sample was incubated in a solution of PSA and a conjugate of antibodies specific to PSA with AuNPs; (**c**) non-specific antibodies were adsorbed in the matrix, then a silicon sample was incubated in a solution of PSA and a conjugate of non-specific antibodies with AuNPs.

**Figure 4:**
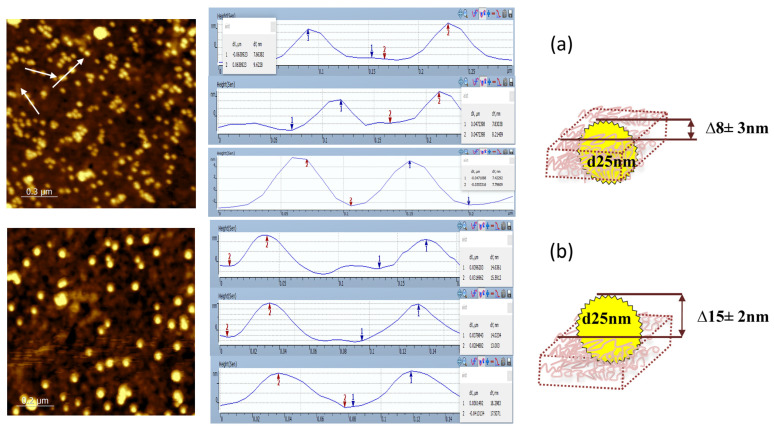

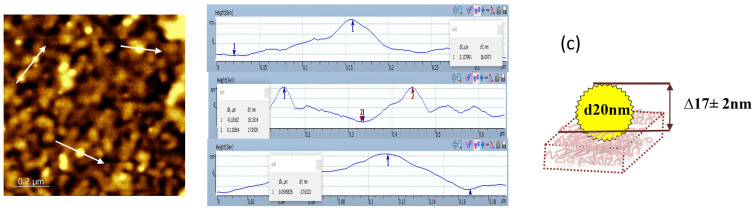
AFM images and surface profiles of silicon samples modified with polyelectrolytes with immune complexes of various compositions. (**a**) antibodies to PSA were adsorbed in the polyelectrolyte matrix, then a silicon sample was incubated in a solution of PSA and a conjugate of antibodies specific to PSA with AuNPs; (**b**) non-specific antibodies were adsorbed in the matrix, then a silicon sample was incubated in a solution of PSA and a conjugate of antibodies specific to PSA with AuNPs; (**c**) non-specific antibodies were adsorbed in the matrix, then a silicon sample was incubated in a solution of PSA and a conjugate of non-specific antibodies with AuNPs.

The processes that take place in the polymer matrix layer during the formation of double and triple sandwich immune complexes labeled with AuNPs were then visualized by applying SEM and AFM methods to a more detailed analysis of three silicon samples (Figure 3 and Figure 4). The use of AuNPs as labels, reliably visualized by SEM and AFM, made it possible to compare the number of immune complexes formed of different antibody/antigen pairs and analyze their location relative to the surface of the nanowire. We compared the effectiveness of the formation of triple sandwich complexes from a PSA molecule and two molecules of specific antibodies, immobilized and labeled with AuNPs; double complexes from a PSA molecule and an antibody molecule labeled with AuNPs (control without immobilized specific antibodies); and non-specific complexes from a PSA molecule and non-specific antibodies labeled with AuNPs (control without immobilized and labeled specific antibodies). In all three studied combinations, we observed the formation of triple and double immune complexes, but their amounts differed significantly.

In order to calculate the number of complexes that were formed, the density of nanoparticles was counted (if each nanoparticle on the surface is part of an immune complex, then the total GNP number is proportional to the total number of complexes formed on the surface). Additionally, the location of the nanoparticles with respect to the silicon surface was examined by splitting the silicon sample.

When two antibody molecules and one antigen molecule combine to form sandwich complexes (Figure 3a), it is evident that AuNPs are “drawn” beneath the PSS layer. This suggests that second antibodies interact with the antigen by penetrating through the PSS layer. Sandwich complexes have a density of 188 AuNPs/μm^2^ and are almost entirely embedded in a polyelectrolyte matrix. It is notable that AuNPs are located at an average distance of 3–5 nm from the silicon surface. As a result, AuNPs are sort of “pulled” beneath the PSS layer. The AFM image (Figure 4a) clearly shows the immersion of nanoparticles in polyelectrolyte matrices; only a small portion of the nanoparticle is still visible above the surface.

When PSA interacts with a matrix with non-specific antibodies (Figure 3b), its molecules enter the matrix and form double complexes of one antigen molecule and one antibody molecule labeled with a nanoparticle. When compared to triple sandwich complexes, we see a less complete partial immersion of AuNPs into the matrix (Figure 3b and Figure 4b). The nanoparticle density (117 AuNPs/μm^2)^ is lower than that for triple sandwich complexes, and they are farther away from the silicon surface (10–15 nm). In this case, the nanoparticles are not covered with a layer of PSS. Thus, the antigen penetrates into the matrix of polyelectrolytes but does not concentrate near the silicon surface. Apparently, it is hindered by a layer of adsorbed, nonspecific antibodies.

In a control experiment using non-specific antibodies and their conjugate with AuNPs, a certain level of non-specific binding was observed: the density of nanoparticles was 11 AuNPs/μm^2^ (Figure 3c). This indicates that non-specific antibodies were adsorbed in small amounts on the surface of the matrix, did not penetrate into the polyelectrolyte layers, and the nanoparticles remained entirely on the surface of the upper PSS layer (Figure 4c).

Our investigation demonstrated the efficiency of antibody adsorption in the 3D matrix containing the layers of oppositely charged polyelectrolytes (PEI and PSS) on the silicon surface. It was discovered that this matrix was permeable to various biomolecules, including PSA, antibodies, and even 25 nm-sized AuNPs. The antigen interacts with antibodies that are specific to it by penetrating the matrix.

The distribution of nanoparticles inside the matrix showed that triple sandwich complexes are located near the surface of the nanowire, while the nanoparticles are drawn into the matrix. In the absence of immobilized specific antibodies, no such effect was observed; double immune complexes were formed at a greater distance from the surface, and the nanoparticles were slightly drawn into the matrix layer. In the control experiment with non-specific antibodies and a conjugate, a small number of nanoparticles remained on the surface of the matrix and were not drawn into it.

Thus, it can be concluded that PSA penetrates into the matrix of polyelectrolytes with specific antibodies and concentrates near the surface. Antibodies immobilized between the layers of polyelectrolytes are able to effectively form complexes with antigens due to the crowding effect, preserving the accessibility and mobility of their active centers. Due to the fact that only immune complexes localized in a thin, near-surface layer of the nanowire are detected by NWFETS, this information is crucial for detecting the antigen using this technique.

### 3.3. Characteristics of NWFETs with Antibodies Adsorbed in the Multilayers of Polyelectrolytes

NW FETs were fabricated as described in detail in the Supplementary Materials. The nanowires were then sequentially modified with layers of polyelectrolyte PEI, PSA-specific antibodies, and polyelectrolyte PSS. In order to improve NW sensitivity to external charges near their surface, the response of the immunosensors was registered in the sub-threshold region characterized by low bias voltage and transport current through the transistors. The regime of low currents is preferable for NW FETs both in terms of low self-noise (current shot noise) and low heating of the NWs [8,18,39].

The operation of the FET with a nanowire channel is based on a change in the conductivity of a semiconductor nanowire in an external electric field, which is formed by any charged particles (ions, molecules, etc.) located on or in the nearest vicinity of the nanowire surface. The surface of the immunosensor is a complex matrix formed by multilayers of charged polyelectrolytes and antibodies, which are also charged under experimental conditions. To test the sensitivity of the nanowire to changes in the electric field, at the first stage we studied its response to changes in proton (hydrogen ion) concentration with changes in the pH of the external environment. A change in pH leads to a change in the concentration of hydrogen ions on the nanowire surface and, as a result, a change in the nanowire conductivity, so the pH sensitivity is an indicator of the transistor sensitivity to any charged particles.

The conductivity of the NW channel depends on the local variation of the field or concentration of hydrogen ions (pH) connecting with the surface of modified silicon. Accordingly [40], the pH sensitivity of the NW FET can be estimated as:

δΨ_0_/δpH = ΔU_ref_/ΔpH

where δΨ_0_ is the change of the insulator–electrolyte potential and U_ref_ is the voltage on the reference electrode.

In order to measure the pH sensitivity of the sensor, the optimal operating point of the NWFET corresponding to the high signal-to-noise ratio of the transistor and a low value of power dissipation in the nanowire channel [18,39] was determined by setting the voltages at the source drain (V_sd_ = 100 mV), the control electrode (V_g_ = 5 V), and the reference electrode (V_ref_ = 450 mV). When the pH value was changed, the voltage on the reference electrode (V_ref_) was adjusted so that the transistor’s current level returned to the value recorded at the prior pH value. Sensitivity to pH was determined as dV_ref_/dpH.

**Figure 5:**
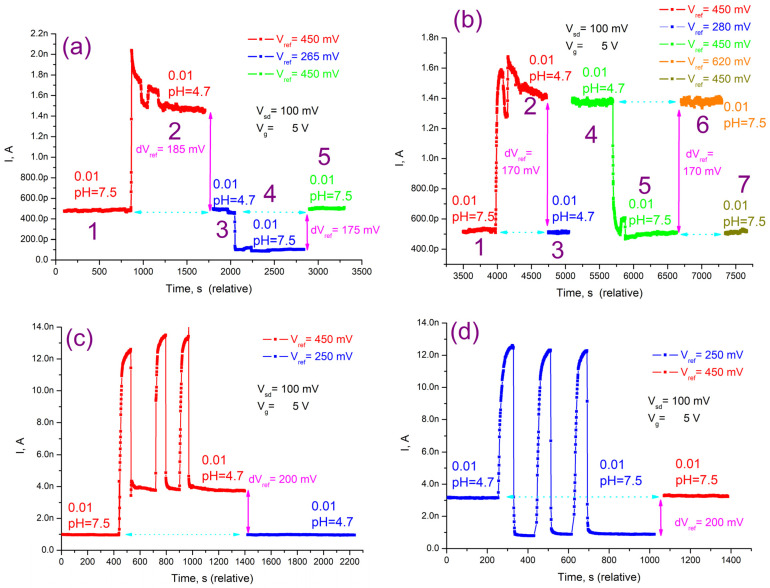
Responses of the NWFETs modified with the layers of polyelectrolyte PEI (**a**,**b**) and a matrix of PEI and PSS with antibodies included (**c**,**d**) to the change in pH. (**b**,**d**) Repeated measurements with a different method of changing pH and V_ref_ for (**a**,**c**), respectively. The current fluctuations (peaks) are caused by replacing a drop of buffer several times with a new one.

The transistor’s current response to a pH change is shown in Figure 5a. At the beginning of the experiment (section a1), the silicon nanowire covered with a PEI layer was placed in a buffer pH 7.5, and the reference electrode voltage was V_ref_ = 450 mV. A change in the pH of the environment led to a change in the conductivity of the nanowire channel and, as a result, to a change in the initial value of the transistor transport current. To determine the sensitivity to pH, the voltage at the reference electrode (V_ref_) was adjusted so that the transistor’s transport current level returned to its initial value.

The transistor’s initial current level was approximately 0.5 nA. The current flowing through the transistor increased to about 1.5 nA when the buffer’s pH dropped to 4.7 units (section a2). The voltage at the reference electrode was decreased from 450 mV to V_ref_ = 265 mV, which corresponds to the initial current level of I~0.5 nA (section a3). Therefore, a 2.8 unit change in pH corresponded to a 185 mV voltage change at the reference electrode, meaning that the pH sensitivity was 66 mV/pH.

Then, the buffer changed back to the original one with pH 7.5, and the level of current decreased to a value of I~0.1 nA (section a4). The voltage at the reference electrode was raised by dV_ref_ = 175 mV to bring it back to the starting level (section a5) and measure the sensitivity at the altered operating point. The sensitivity to pH in this case matched a value of 62 mV/pH.

A slightly different approach was applied during the repeated measurements (Figure 5b). Sections b1, b2, and b3 were measured using the same methodology as in the prior case, but the dV_ref_ change was 170 mV by 2.8 pH units (61 mV/pH). Then, the voltage at the reference electrode returned to the initial level V_ref_ = 450 mV (section b4) and at this operating point the buffer changed to the initial one with pH = 7.5, which led to a decrease in the transistor current to the initial value I~0.5 nA (section b5). The voltage at the reference electrode was raised to V_ref_ = 620 mV in order to return to the signal level that corresponded to pH = 4.7 and V_ref_ = 450 mV (section b6). In this case, the dV_ref_ change was likewise 170 mV, which corresponded to a sensitivity to pH of 68 mV/pH. The current level was restored to the initial level of I~0.5 nA by lowering the V_ref_ in reverse to 450 mV (section b7). The error in the pH measurements, determined mainly by the drift of the current line, was ±3 mV/pH, so the measured values can be considered the same.

Measurements of the nanowire covered by PEI and PSS matrix with antibodies adsorbed between them (Figure 5c,d) were performed in the same manner; the change in V_ref_ (200 mV by 2.8 pH units) corresponds to a pH change of 71 mV/pH.

As a result, the pH sensitivity of the nanowires containing antibodies adsorbed in the polyelectrolyte matrix corresponds to a value of 71 ± 3 mV/pH, which is higher than the value of 60 mV/pH observed when silicon was treated with silanes [8].

### 3.4. Detection of PSA on the NWFETs

The determination of PSA on NW FETs is based on the direct binding of the antigen with specific antibodies immobilized in the complex matrix formed by multilayers of charged polyelectrolytes on the surface of a thin nanowire channel (Figure 1). During the formation of immune complexes of charged antibody and antigen molecules, electric charges are redistributed near the surface of the nanowire, which led to a change in the nanowire conductivity. The specificity and selectivity of PSA determination are ensured by the properties of monoclonal antibodies. The high specificity of this antibody clone against PSA was characterized earlier in the development of a standard ELISA method and the FETs with nanowires modified with gold nanoparticles using a collection of clinical samples [8].

**Figure 6:**
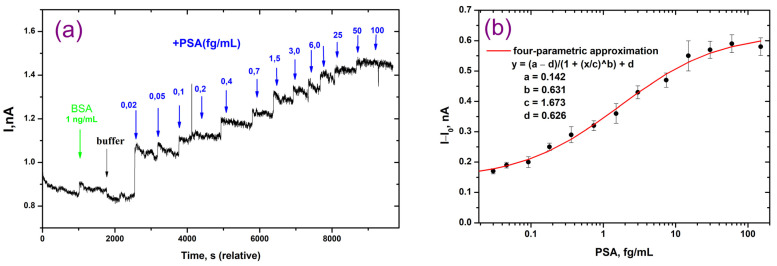
Determination of PSA on the NWFETs modified by a matrix of charged polyelectrolytes. (**a**) Transistor responses after adding buffer, BSA solution, and PSA solutions with different concentrations; (**b**) Calibration curve for the detection of PSA in 0.01 × PBS buffer.

The performance of the NWFETs at various PSA concentrations is shown in Figure 6. It can be seen that as the PSA concentration increases, the transport current increases as well. Even at a PSA concentration as low as 0.02 fg/mL, there is already a noticeable and substantial increase in the transport current (Figure 6a). About 10 s were needed to reach the maximum response to antigen solutions, and the signal remained steady for at least a few minutes. The analysis time per sample was 1 min, which included signal detection and washing. The control experiment with BSA (1 μg/mL) showed no detectable shift in the current of the NW FETs above the noise level.

The calibration curve for the detection of PSA in a 0.01 × PBS buffer is presented in Figure 6b. The calibration curve demonstrated a direct relationship between response and concentration of analyte. It was nonlinear, which is typical for immunoassay methods. In order to fit the curve, we used a 4-parameter mathematical model. The method is characterized by an ultra-low detection limit of 0.04 fg/mL (1.2 aM). For PSA determination in a buffer, the dynamic range is 0.08 fg/mL to 60 fg/mL.

Table 2 presents a comparison of the new approach with several electrochemical immunosensors for PSA detection that have been proposed recently. For comparison, biosensors based on NWFETs, FETs, and several variants of electrodes (screen-printed and gold) coated with films of various compounds, including polymers, were selected. Analysis of the analytical characteristics of biosensors shows that NWFETs are characterized by the lowest detection limit of PSA (this work, [41]). Between them, our technique with multilayer polyelectrolyte-modified NWs achieved an ultra-low PSA detection limit. Modification of imunosensors with different films, including polymers, improves their sensitivity [42,43,44,45]. Several immunosensors [42,43] showed comparable LOD with the NWFETs modified with APTES and glutaraldehyde [41]. It should be noted that for these immunosensors, one type of electrode coating was used. In contrast, in our immunosensor, antibodies were immobilized between the layers of oppositely charged polymers. As a result, we were able to reduce the detection limit of PSA by several orders of magnitude using monoclonal antibodies of the same clone compared to the NWFETs, in which silicon was modified by AuNPs [8]. This fact demonstrates how the polyelectrolyte matrix preserves the immunological characteristics of antibodies and has a positive impact on their properties.

At the same time, the dynamic range of detectable PSA concentrations by our immunosensor is three orders of magnitude and is not as wide as in other techniques. The possibility of dilution of the serum sample makes it possible to reduce the potential interference of biological samples. Despite the fact that the range of three orders of magnitude meets the diagnostic requirements, further research is needed to examine the range in biological samples.

The analysis of the applicability of various electrochemical immunosensor technologies for POC is of great practical interest because PSA detection is valuable for early screening of prostate cancer and monitoring recurrence after treatment. The criteria for POC systems involve rapid assay time, measuring to be performed at the place of the patient, minimal sample preparation, small sample volume, automation by a portable device, and fitting the sensitivity and concentration range [49]. At present, commercial POC systems for PSA detection are based on lateral-flow immunoassays with colorimetric detection. They require little sample volume and are quick, but they do not always fulfill the criteria for sensitivity, specificity, and measurement range. It is believed that electrochemical biosensors better match POC requirements. The immunosensors reported in the literature exhibit promising results and have good potential for ensuring low detection limits, small sample volumes, and automation capabilities. At the same time, only some immunosensors have been validated using real serum samples [44,45,46]. Our approach and a few other novel principles of PSA determination have only been evaluated with PSA samples in buffer systems. Further studies are required to investigate the long-term stability of the immunosensors, explore the need to expand the dynamic range, and validate them with serum samples.

## 4. Conclusions

In this work, we have demonstrated the advantages of coating silicon nanowires with layers of charged polyelectrolytes. Together, they form a three-dimensional matrix that is permeable to biomolecules and efficiently absorbs antibodies. Multilayers of polyelectrolytes provide a polyelectrolyte-protein “crowding” medium that promotes the preservation of antibody properties and contributes to the concentration of antigen in the near-surface layer. As a result, NWFETs with antibodies in a polyelectrolyte matrix show a high sensitivity to pH changes, which significantly exceeds the theoretical limit of Nernst. The immunosensor is characterized by the lowest PSA detection limit in buffer systems to date. Coating the NW surface with multiple polymer layers significantly simplifies the process of preparing the sensor surface compared to the chemical modification of silicon for the covalent attachment of antibodies. The sensor takes less than a minute, and the measurement is performed in real-time. This approach seems promising for further investigation of its applicability for early screening of prostate cancer and POC systems. Future research will focus on optimizing immunosensors for measuring PSA in biological samples, examining their long-term stability, and validating them with real serum samples.

## Figures and Tables

**Table 1:** Characteristics of the matrix on the silicon surface during sequential application of polyelectrolytes, antibodies, and antigen.

Components of the Matrix	Matrix Thickness Measured by SEM, nm	Matrix Thickness Measured by AFM, nm	Surface Roughness Measured by AFM, nm
No matrix (native silicon with a layer of silicon oxide)	3 ± 0.5	ND	0.5
PEI	6 ± 3	10 ± 2	3.7
PEI, AB/_PSA_, PSS	22 ± 7	23 ± 5	3.7
(PEI, AB/_PSA_, PSS) after interacting with PSA	27 ± 10	32 ± 5	7.8
(PEI, AB/_PSA_, PSS) after interacting with PSA and Ab/_PSA_–AuNPs	25 ± 3 *	35 ± 5	5.0
Control: (PEI, AB/_non-specific_, PSS) after interacting with PSA and Ab/_PSA_–AuNPs	20 ± 5 *	37 ± 5	5.3
Control: (PEI, AB/_non-specific_, PSS) after interacting with PSA and Ab/_non-specific_–AuNPs	20 ± 5 *	24 ± 5	3.3

* The thickness of the matrix layer was measured without accounting for nanoparticle size.

**Table 2:** Analytical performances of different electrochemical immunosensors developed for quantitative determination of PSA.

Biosensor Type/Surface Modification	Limit of PSA Detection, fg/mL	Detection Range, fg/mL	Reference
Si NWFETs//matrix of polyelectrolytes PEI and PSS	0.04 (1.2 aM)	0.06–60	This study
Si NWFETs//GOPS-SH and AuNPs//the same clone of antibodies to PSA	23	23–5 × 10^8^	[8]
n- and p-type Si NW FET arrays//APTES and glutaraldehyde	1.0	1.0–10^5^	[41]
AlGaN/GaN HEMT//ethanolamine	1 (in PBS buffer)	1–10^8^ (1 fg/mL to 100 ng/mL).	[42]
Electrochemical capacitive immunosensor/gold electrode modified with a thin film of isophthalic acid	3.3 (3.3 × 10^−6^ ng/mL)	1–10^8^ (1 × 10^−5^−100 ng/mL)	[43]
Immunosensor//screen-printed electrodes//AgNPs/ds- DNA/PEDOT	17	100–10^8^ (0.1 pg/mL–100 ng/mL)	[44]
Electrochemical immunosensor with silica nanochannel films//GPTMS	100 0.1 pg/mL	10^3^–10^8^ (1 pg/mL to 100 ng/mL)	[45]
Graphene FET with coplanar electrodes//glutaraldehyde	10^3^ (1 pg/mL in serum)	Up to 4 × 10^7^ (up to 4 ng/mL in 100 mM phosphate buffer)	[46]
A dual-gate PEG-modified NR-ISFET nanoribbon-based ion-sensitive field-effect transistor (NR-ISFET)//polyethylene glycol	10 pM (in 100 mM phosphate buffer)	10 pM to 1 μM (in 100 mM phosphate buffer)	[47]
Si NW-Ionsensitive field-effect transistor (Si-NW ISFET)//DNA aptamer	ND *	10^3^–10^9^ (1 pg/mL to 1 μg/mL)	[48]

* Not determined.

## Data Availability

Data are contained within the article and Supplementary Materials.

## Supplementary Materials

The following supporting information can be downloaded: https://www.mdpi.com/article/10.3390/polym16030332/s1, Figure S1: Dependence of the thickness of the PEI layer on the silicon surface on its concentration; Figure S2: Thickness of the PEI layer (a) and the matrix layer of polyelectrolytes with antibodies (b) for different silicon samples; Figure S3: Main fabrication steps of a transistor with a nanowire channel. (a)—formation of an aluminum mask; (b)—transfer of the mask pattern to the top layer of silicon using RIE; (c)—formation of metallic contact pads; (d)—isolation of current-carrying elements of the structure from contact with the liquid medium; Figure S4: SEM images of an aluminum mask on the top of the active silicon layer (a) and the structure of a nanowire transistor with contact pads covered by dielectric layers (b); Figure S5: Transistor transport current vs. main gate voltage V_g_ (top axis) and reference electrode voltage V_ref_ (bottom axis). References [33,34,39] are cited in the supplementary materials.

## References

[1] Sadighbayan D., Hasanzadeh M., Ghafar-Zadeh E. (2020). Biosensing based on field-effect transistors (FET): Recent progress and challenges. Trends Analyt. Chem..

[2] Cui Y., Lieber C.M. (2001). Functional nanoscale electronic devices assembled using silicon nanowire building blocks. Science.

[3] Salfi J., Savelyev I.G., Blumin M., Nair S.V., Ruda H.E. (2010). Direct observation of single-charge detection capability of nanowire field-effect transistors. Nat. Nanotech..

[4] Clement N., Nishiguchi K., Dufreche J.F., Guerin D., Fujiwara A., Vuillaume D. (2011). A silicon nanowire ion-sensitive field-effect transistor with the elementary charge sensitivity. Appl. Phys. Lett..

[5] Wang Z., Lee S., Koo K.-I., Kim K. (2016). Nanowire-Based Sensors for Biological and Medical Applications. IEEE Trans. NanoBiosci..

[6] Panahi A., Sadighbayan D., Forouhi S., Ghafar-Zadeh E. (2021). Recent Advances of Field-Effect Transistor Technology for Infectious Diseases. Biosensors.

[7] Malsagova K.A., Ivanov Y.D., Pleshakova T.O., Kaysheva A.L., Shumov I.D., Kozlov A.F., Archakov A.I., Popov V.P., Fomin B.I., Latyshev A.V. (2015). A SOI-nanowire biosensor for the multiple detection of D-NFATc1 protein in the serum. Anal. Methods.

[8] Presnova G., Presnov D., Krupenin V., Grigorenko V., Trifonov A., Andreeva I., Ignatenko O., Egorov A., Rubtsova M. (2017). Biosensor based on a silicon nanowire field-effect transistor functionalized by gold nanoparticles for the highly sensitive determination of prostate specific antigen. Biosens. Bioelectron..

[9] Zhao W., Hu J., Liu J., Li X., Sun S., Luan X., Zhao Y., Wei S., Li M., Zhang Q. (2022). Si nanowire Bio-FET for electrical and label-free detection of cancer cell-derived exosomes. Microsyst. Nanoeng..

[10] Seo G., Lee G., Kim M.J., Baek S.H., Choi M., Ku K.B., Lee C.S., Jun S., Park D., Kim H.G. (2020). Rapid Detection of COVID-19 Causative Virus (SARS-CoV-2) in Human Nasopharyngeal Swab Specimens Using Field-Effect Transistor-Based Biosensor. ACS Nano.

[11] Kim K., Park C., Kwon D., Kim D., Meyyappan M., Jeon S., Lee J.S. (2016). Silicon nanowire biosensors for detection of cardiac troponin I (cTnI) with high sensitivity. Biosens. Bioelectron..

[12] Espinosa F.M., Uhlig M.R., Garcia R. (2022). Molecular Recognition by Silicon Nanowire Field-Effect Transistor and Single-Molecule Force Spectroscopy. Micromachines.

[13] Hu Q., Chen S., Solomon P., Zhang Z. (2021). Ion sensing with single charge resolution using sub–10-nm electrical double layer–gated silicon nanowire transistors. Sci. Adv..

[14] Wasfi A., Awwad F., Gelovani J.G., Qamhieh N., Ayesh A.I. (2022). COVID-19 Detection via Silicon Nanowire Field-Effect Transistor: Setup and Modeling of Its Function. Nanomaterials.

[15] Abidin W.A.B.Z., Nor N.M.M., Arshad M.K.M., Fathil M.F.M., Parmin N.A., Sisin N.A.H.T., Ibau C., Azlan A.S. (2022). Femtomolar Dengue Virus Type-2 DNA Detection in Back-gated Silicon Nanowire Field-effect Transistor Biosensor. Curr. Nanosci..

[16] Zafar S., D’Emic C., Jagtiani A., Kratschmer E., Miao X., Zhu Y., Mo R., Sosa N., Hamann H., Shahidi G. (2018). Silicon Nanowire Field Effect Transistor Sensors with Minimal Sensor-to-Sensor Variations and Enhanced Sensing Characteristics. ACS Nano.

[17] Kesler V., Murmann B., Soh H.T. (2020). Going beyond the Debye Length: Overcoming Charge Screening Limitations in Next-Generation Bioelectronic Sensors. ACS Nano.

[18] Presnov D.E., Bozhev I.V., Miakonkikh A.V., Simakin S.G., Trifonov A.S., Krupenin V.A. (2018). Local sensor based on nanowire field effect transistor from inhomogeneously doped silicon on insulator. J. Appl. Phys..

[19] Rajan N.K., Routenberg D.A., Reed M.A. (2018). Optimal signal-to-noise ratio for silicon nanowire biochemical sensors. Appl. Phys. Lett..

[20] De Vico L., Sorensen M.H., Iversen L., Rogers D.M., Sorensen B.S., Brandbyge M., Nygard J., Martinez L., Jensen J.H. (2011). Quantifying signal changes in nano-wire based biosensors. Nanoscale.

[21] Li J., Zhang Y., To S., You L., Sun Y. (2011). Effect of nanowire number, diameter, and doping density on nano-FET biosensor sensitivity. ACS Nano.

[22] Stern E., Wagner R., Sigworth R.J., Breaker R., Fahmy T.M., Reed M.A. (2007). Importance of the Debye screening length on nanowire field effect transistor sensors. Nano Lett..

[23] Awsiuk K., Budkowski A., Psarouli A., Petrou P., Bernasik A., Kakabakos S., Rysz J., Raptis I. (2013). Protein adsorption and covalent bonding to silicon nitride surfaces modified with organo-silanes: Comparison using AFM, angle-resolved XPS and multivariate ToF-SIMS analysis. Colloids Surf. B Biointerfaces.

[24] Ghobaei Namhil Z., Kemp C., Verrelli E., Iles A., Pamme N., Adawi A.M., Kemp N.T. (2019). A label-free aptamer-based nanogap capacitive biosensor with greatly diminished electrode polarization effects. Phys. Chem. Chem. Phys..

[25] Gao N., Zhou W., Jiang X., Hong G., Fu T.-M., Lieber C.M. (2015). General Strategy for Biodetection in High Ionic Strength Solutions Using Transistor-Based Nanoelectronic Sensors. Nano Lett..

[26] Becker A.L., Henzler K., Welsch N., Ballauff M., Borisov O. (2012). Proteins and polyelectrolytes: A charged relationship. Curr. Opin. Colloid Interface Sci..

[27] Lee A.A., Kostinski S.V., Brenner M.P. (2018). Controlling Polyelectrolyte Adsorption onto Carbon Nanotubes by Tuning Ion–Image Interactions. J. Phys. Chem. B.

[28] Stekolshchikova A.A., Radaev A.V., Orlova O.Y., Nikolaev K.G., Skorb E.V. (2019). Thin and Flexible Ion Sensors Based on Polyelectrolyte Multilayers Assembled onto the Carbon Adhesive Tape. ACS Omega.

[29] Zhao H., Ibrahimova V., Garanger E., Lecommandoux S. (2020). Dynamic Spatial Formation and Distribution of Intrinsically Disordered Protein Droplets in Macromolecularly Crowded Protocells. Angew. Chem. Int. Ed..

[30] Baldina A.A., Nikolaev K.G., Ivanov A.S., Nikitina A.A., Rubtsova M.Y., Vorovitch M.F., Ishmukhametov A.A., Egorov A.M., Skorb E.V. (2022). Immunochemical Biosensor for Single Virus Particle Detection Based on Crowding Molecular Polyelectrolyte System. J. Appl. Polym. Sci..

[31] Frens G. (1973). Controlled nucleation for the regulation of the particle size in monodisperse gold suspensions. Nat. Phys. Sci..

[32] Green C.P., Lioe H., Cleveland J.P., Proksch R., Mulvaney P., Sader J.E. (2004). Normal and torsional spring constants of atomic force microscope cantilevers. Rev. Sci. Instr..

[33] Tsiniaikin I.I., Presnova G.V., Bozhev I.V., Skorik A.A., Rubtsova M.Y., Kamalov A.A., Matskeplishvili S.T., Snigirev O.V., Krupenin V.A., Presnov D.E. (2020). A sensor system based on a field-effect transistor with a nanowire channel for the quantitative determination of thyroid-stimulating hormone. Mosc. Univ. Phys. Bull..

[34] Presnova G.V., Tcinyaykin I.I., Bozhev I.V., Rubtsova M.Y., Shorokhov V.V., Trifonov A.S., Ulyashova M.M., Krupenin V.A., Presnov D.E. (2019). Thyroglobulin detection by biosensor based on two independent Si NWFETs. Proc. SPIE-Int. Soc. Opt. Eng..

[35] Soranno A., Koenig I., Borgia M.B., Hofmann H., Zosel F., Nettels D., Schuler B. (2014). Single-molecule spectroscopy reveals polymer effects of disordered proteins in crowded environments. Proc. Natl. Acad. Sci. USA.

[36] Filoti D.I., Shire S.J., Yadav S., Laue T.M. (2015). Comparative study of analytical techniques for determining protein charge. J. Pharm. Sci..

[37] Füzik T., Formanov P., Ružek D., Yoshii K., Niedrig M., Plevka P. (2018). Structure of tick-borne encephalitis virus and its neutralization by a monoclonal antibody. Nat. Commun..

[38] Straeten A.V., Bratek-Skicki A., Jonas A.M., Fustin C.-A., Dupont-Gillain C. (2018). Integrating Proteins in Layer-by-Layer Assemblies Independently of their Electrical Charge. ACS Nano.

[39] Trifonov A.S., Presnov D.E., Bozhev I.V., Evplov D.A., Desmaris V., Krupenin V.A. (2017). Non-contact scanning probe technique for electric field measurements based on nanowire field-effect transistor. Ultramicroscopy.

[40] Van Hal R.E.G., Eijkel J.C.T., Bergveld P. (1995). A novel description of ISFET sensitivity with the buffer capacity and double-layer capacitance as key parameters. Sens. Actuators B.

[41] Gao A., Lu N., Dai P., Fan C., Wang Y., Li T. (2014). Direct ultrasensitive electrical detection of prostate cancer biomarkers with CMOS-compatible n- and p-type silicon nanowire sensor arrays. Nanoscale.

[42] Gu Z., Wang J., Miao B., Zhao L., Liu X., Wu D., Li J. (2019). Highly sensitive AlGaN/GaN HEMT biosensors using an ethanolamine modification strategy for bioassay applications. RSC Adv..

[43] Mwanza D., Adeniyi O., Tesfalidet S., Nyokong T., Mashazi P. (2022). Capacitive Label-Free Ultrasensitive Detection of PSA on a Covalently Attached Monoclonal Anti-PSA Antibody Gold Surface. J. Electroanal. Chem..

[44] Liu Y. (2023). Highly sensitive sensing detection of prostate-specific antigen based on point-of-care electrochemical immunosensor. Int. J. Electrochem. Sci..

[45] Ma K., Zheng Y., An L., Liu J. (2022). Ultrasensitive Immunosensor for Prostate-Specific Antigen Based on Enhanced Electrochemiluminescence by Vertically Ordered Mesoporous Silica-Nanochannel Film. Front. Chem..

[46] Mandal N., Pakira V., Samanta N., Das N., Chakraborty S., Pramanick B., RoyChaudhuri C. (2021). PSA Detection using Label Free Graphene FET with Coplanar Electrodes Based Microfluidic Point of Care Diagnostic Device. Talanta.

[47] Ma S., Li X., Lee Y.K., Zhang A. (2018). Direct Label-free Protein Detection in High Ionic Strength Solution and Human Plasma Using Dual-Gate Nanoribbon-based Ion-Sensitive Field-Effect Transistor Biosensor. Biosens. Bioelectron..

[48] Rani D., Pachauri V., Madaboosi N., Jolly P., Vu X.T., Estrela P., Chu V., Conde J.P., Ingebrandt S. (2018). Top-Down Fabricated Silicon Nanowire Arrays for Field-Effect Detection of Prostate-Specific Antigen. ACS Omega.

[49] Garg S., Sachdeva A., Peeters M., McClements J. (2023). Point-of-Care Prostate Specific Antigen Testing: Examining Translational Progress toward Clinical Implementation. ACS Sens..

