# Supplementary material for: Ultrasensitive Detection of PSA Using Antibodies in Crowding Polyelectrolyte Multilayers on a Silicon Nanowire Field-Effect Transistor
Source: Polymers (Basel). 2024 Jan 25;16(3):332. doi: 10.3390/polym16030332 (PMC10857411; doi:10.3390/polym16030332)
Supplement: Supplementary file 1 [file polymers-16-00332-s001.zip › polymers-2755894-supplementary.pdf]

Supplementary Materials

Ultrasensitive Detection of PSA Using Antibodies in Crowding Polyelectrolyte Multilayers on a Silicon Nanowire Field-Effect Transistor

Galina V. Presnova [1], Denis E. Presnov [2,3,*], Mariya M. Ulyashova [1], Ilia I. Tsiniaikin [2], Artem S. Trifonov [2], Ekaterina V. Skorb [4], Vladimir A. Krupenin [2], Oleg V. Snigirev [2] and Maya Yu. Rubtsova [1,*]

1 Department of Chemistry, Lomonosov Moscow State University, 119991 Moscow, Russia
2 Faculty of Physics, Lomonosov Moscow State University, 119991 Moscow, Russia
3 D.V. Skobeltsyn Institute of Nuclear Physics, Lomonosov Moscow State University, 119991 Moscow, Russia
4 Infochemistry Scientific Center of ITMO University, 191002 Saint Petersburg, Russia
* Correspondence: denis.presnov@physics.msu.ru (D.E.P.); myr@enz.chem.msu.ru (M.Y.R.)

S1.

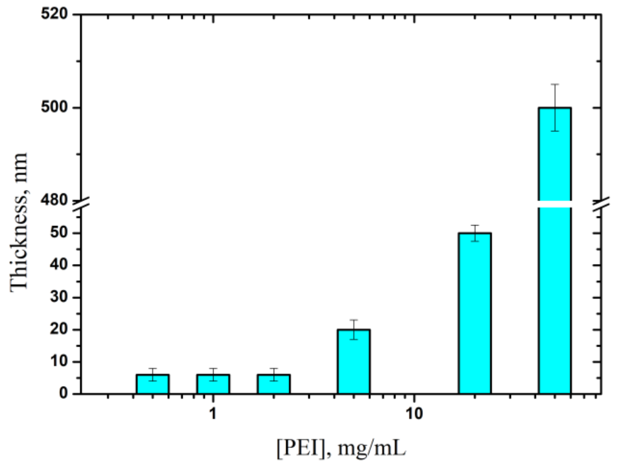

Figure S1. Dependence of the thickness of the PEI layer on the silicon surface on its concentration.

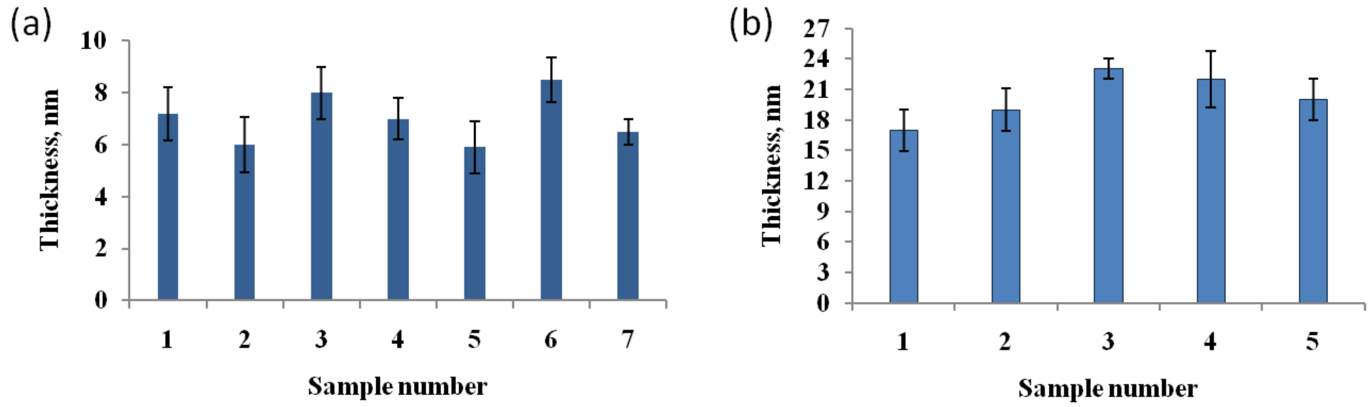

Figure S2. Thickness of the PEI layer (a) and the matrix layer of polyelectrolytes with antibodies (b) for different silicon samples. The relative deviation was calculated from 10 measurements.

S2. Fabrication of NW FETs

The fabrication technique of solid-state sensor structures is based on the "top-down" reactive ion etching (RIE) process of the top layer of silicon-on-insulator (SOI) wafers through a mask formed by electron beam lithography [1,2]. The technique is fully CMOS compatible and also involves thin film vacuum deposition of dielectric and metallic materials. The main stages of the basic operations for the fabrication of transistors with a nanowire channel are presented in Figure S3.

For the experimental samples fabrication, we used SOI wafers from Soitec company, created using UniBond® technology with the 110 nm thick top layer of silicon (crystal orientation <100>, doping - p-type/boron, resistivity 8.5 - 11.5 Ohm*cm). The massive (750 μm thick) supporting handle wafer had the same parameters and was separated from the active top layer by a 200 nm thick SiO_2 buried oxide layer. The handle wafer later was used as a bottom gate to adjust the working point of the transistor.

Before starting the process, the wafer surface was carefully cleaned in organic solvents (acetone, isopropanol). Complete chemical cleaning was provided by using a 6% buffered ammonium hydrofluoride solution NH_4HF_2 that was removed in deionized water for 1 minute. After this, the sample was kept in a dry and clean atmosphere for 24 hours until a native oxide formed on the silicon surface.

The mask of a transistor structures was formed on the surface of active top monocrystalline silicon layer by electron-beam lithography, and metal vacuum deposition techniques. In the central regions of the chip a nanowire pattern 80–90 nm wide and 3–5 μm long was drawn in a thin layer of positive electron beam resist PMMA 950K (MCC A4) using a Carl Zeiss "Supra-40" field emission electron microscope equipped with an "Elphy Quantum" Raith lithography attachment. The exposure (15 nm) layer of aluminum was deposited in open windows of the resist with the 0.3 nm/s rate at base pressure of $4x10^{-7}$ mbar. The unexposed areas of PMMA resist covered by waster aluminum were removed by a lift-off process in acetone. As a result, an aluminum mask corresponding to the desired pattern remains on the surface of the sample (Figure S3a, Figure S4a).

The transfer of the transistor pattern to the active silicon layer was carried out using the process of anisotropic reactive ion etching (RIE) in fluorine-containing plasma (SF_6, 0.2 Pa, 50

W, RDE-300-Alcatel): the areas of the silicon layer not covered by an aluminum mask were completely removed to the underlying SiO_2 layer. The duration of the process was controlled by laser reflecto-interferometry technique using the Multisem-440 diagnostic complex and averaged about 50 seconds (Figure S2b). The remaining part of the Al mask was removed in a weakly alkaline KOH solution (2%).

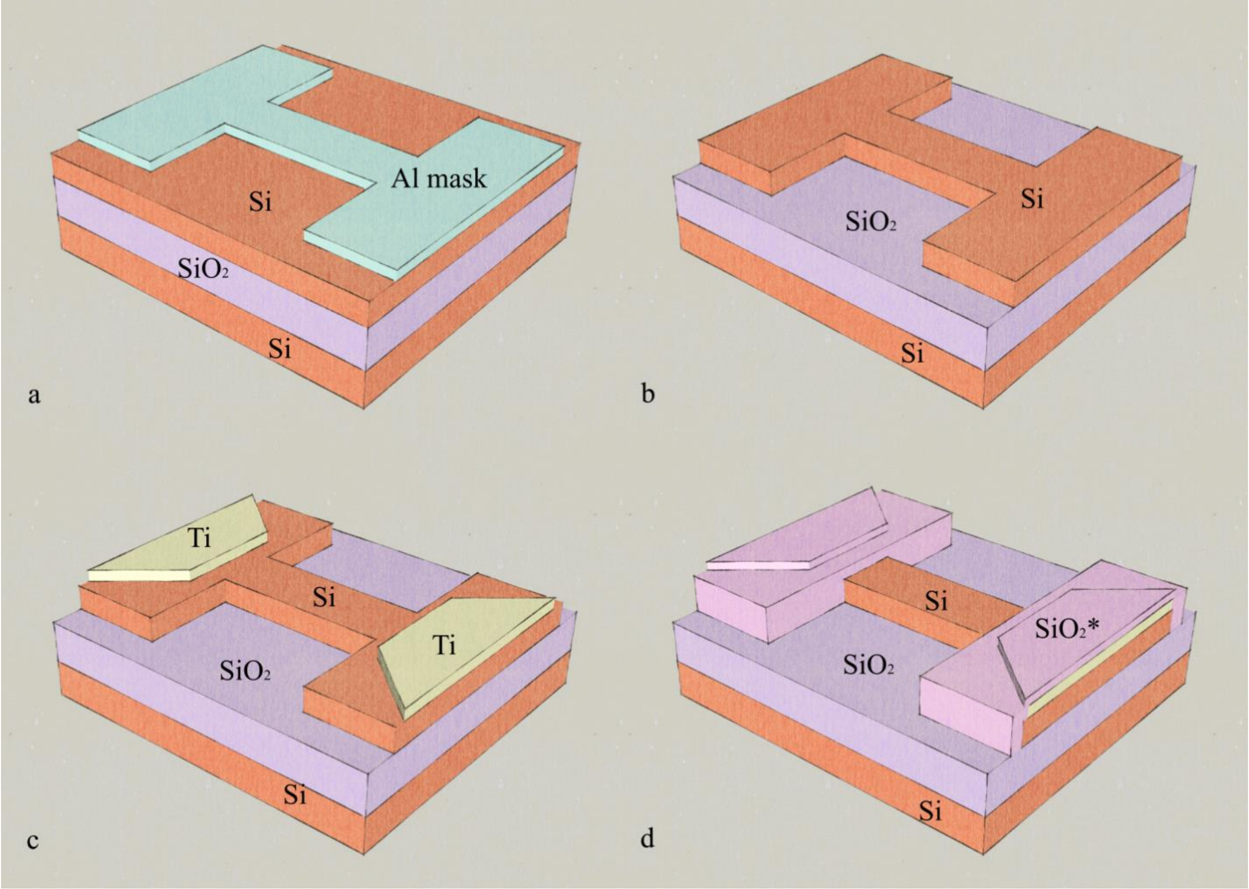

Figure S3. Main fabrication steps of a transistor with a nanowire channel. (a) – formation of an aluminum mask; (b) - transfer of the mask pattern to the top layer of silicon using RIE; (c) - formation of metallic contact pads; (d) – isolation of current-carrying elements of the structure from contact with the liquid medium.

a) b)

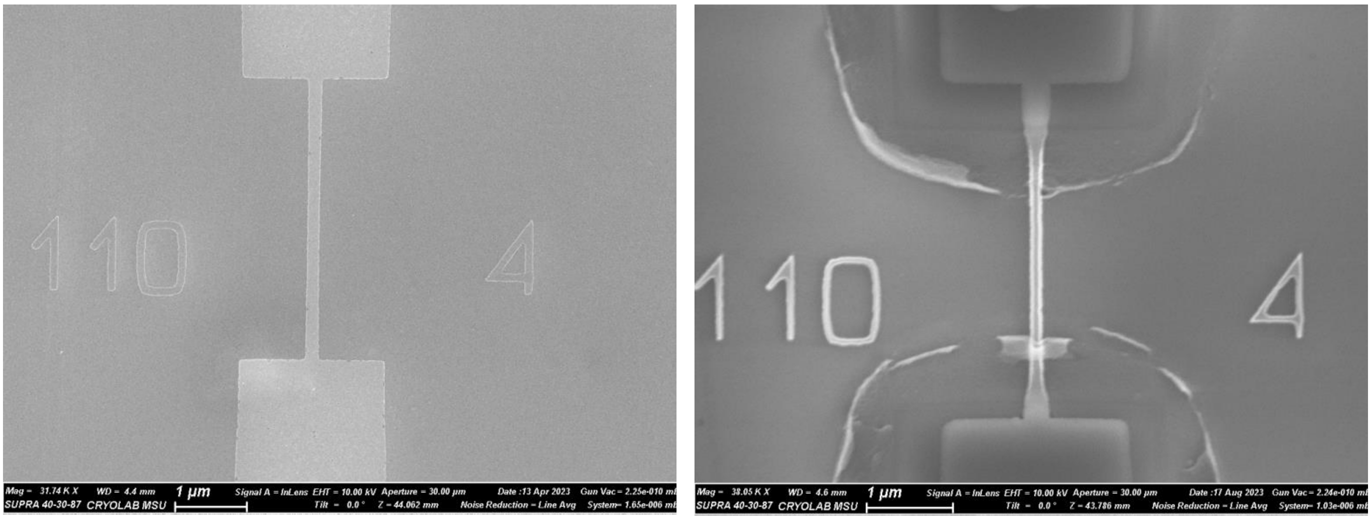

Figure S4. SEM images of aluminum mask on the top of active silicon layer (a) and the structure of a nanowire transistor with contact pads covered by dielectric layers (b).

dose was 300 μC/cm^2 at an accelerating voltage of 20 kV. The resist was then developed in a mixture of IPA and DI water at a ratio of 97% by volume. Using electron beam evaporator (Leybold, L-560), a thin

To reliably isolate the contact pads from the conductive silicon substrate, the thickness of the dielectric layer was increased over the entire surface of the sample, with the exception of its central part. Thickening was carried out by three successive deposition of SiO2 layers using high-frequency (RF) magnetron sputtering (O_2 plasma, 2x10^{-3} mbar, 200 W, Z-400-LeyBold) at a speed of about 0.2 nm/s. The thickness of each layer was 200 nm. Before each deposition, a two-layer PMMA-MAA/PMMA resistive mask with a total thickness of ~700 nm was formed on the sample surface using optical photolithography in the deep ultraviolet range (λ = 200 nm).

The contact pads pattern was determined by both optical and electron beam lithography using a two-layer positive PMMA/MAA resist, as in the previous case. Large areas were exposed optically using an appropriate quartz photomask. In the central part of the chip (~100 μm^2), e-beam lithography was used, allowing precise (~30 nm) alignment with the already formed high-resolution pattern of silicon nanowires (Figure S2c).Titanium contact pads to the silicon layer were formed by magnetron sputtering (Ar plasma, 1.2x10^{-2} mbar, 400 W, Z-400-

LeyBold). At the point of contact of the metal with slightly doped silicon, Schottky barriers are formed, having the value of 0.28 eV in the case of electronic conductivity [3].

Finally, the entire surface of the chip, with the exception of central regions with nanowires and a contact pads at the edges was covered with two layers of SiO_2 dielectric layer 200 nm thick in the manner described above. For each layer, a separate resist mask was prepared using optical and e-beam techniques. The formed pattern completely covered all open metal areas of the contact pads, and each subsequent layer was covering a larger area (Figure S3d, Figure S4b). This special care was taken to ensure reliable isolation of the titanium conductive leads from reaction liquid medium

The top view of the nanowire transistor structure were obtained using a scanning electron microscope and shown in Figure S3b. In each central part, 6 transistors were fabricated with a nanowire length of 3–5 μm and a width of 80–90 nm.

For measurements, the wafers were splitted into separate parts, and each sample was fixed on a special ceramic holder. The contacts of the holder were connected using ultrasonic bonding with an aluminum wire (~ 30 μm, West Bond -7476) with areas of the contact pads on the chip not covered by the dielectric. A silicone sealant was used for isolation of open wires and contacts from liquid. At the same time, work areas with the transistors remained uncovered.

S3. Electrical characterization of NW FETs

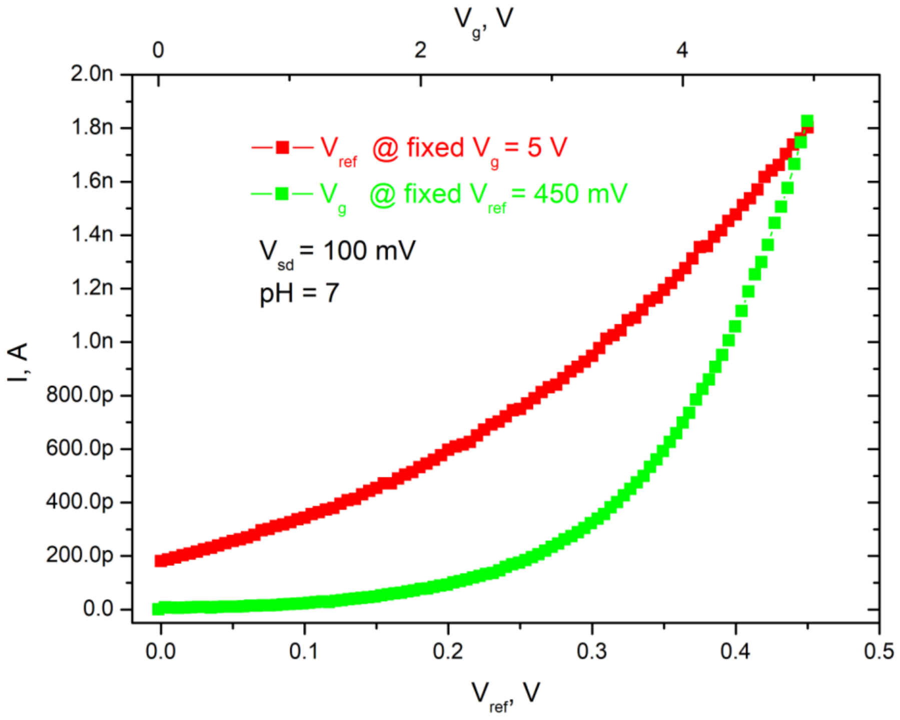

Figure S5. Transistor transport current vs. main gate voltage V_g (top axis) and reference electrode voltage V_{ref} (bottom axis).

Measurements of the transistor transport characteristics, as well as measurements of the NWFET responses based on them, were carried out using a home-made measuring system consisting of a preamplifier, a voltage setting unit and an interface unit providing communication with the control computer. The preamplifier's own low noise level (30 fA/√Hz) made it possible to register ultra-low current changes up to picoamps.

The electrical characterization of the fabricated structures was done by measuring their current dependences in a neutral liquid environment. The supporting silicon wafer was played the role of the main gate (V_g), and the AgCl reference electrode, immersed in the solution drop, served as an additional gate (V_{ref}).

The optimal operating point, corresponding the high signal-to-noise ratio of the transistor and a low value of power dissipation in the nanowire channel [4], was fixed at the voltage V_{sd} = 100 mV between the electrodes, which also provided a low level of transport current (2-4 nA) through a nanowire (see also [1,2]).

Measurements were performed at positive values of V_g and V_{ref} voltages, which correspond to the inverse (electronic) conductivity channel of the transistor.

Figure S5 shows the characteristic dependences of the transistor transport current from the voltages V_{ref} and V_g at the control electrodes. Analytical characteristics were recorded at V_{sd} = 100 mV, V_g = 5 V; V_{ref} = 450 mV in a microfluidic system with static mode, without fluid flow, which provided stable and reproducible response signals.

References

1. Tsiniaikin, I.I.; Presnova, G.V.; Bozhev, I.V.; Skorik, A.A.; Rubtsova, M.Yu; Kamalov, A.A.; Matskeplishvili, S.T.; Snigirev, O.V.; Krupenin, V.A.; Presnov, D.E. A sensor system based on a field-effect transistor with a nanowire channel for the quantitative determination of thyroid-stimulating hormone. *Mosc. Univ. Phys. Bull.* **2020**, *75*, 645–656. https://doi.org/10.3103/s002713492006020x.
2. Presnova, G.V.; Tcinyaykin, I.I.; Bozhev, I.V.; Rubtsova, M.Y.; Shorokhov, V.V.; Trifonov, A.S.; Ulyashova, M.M.; Krupenin, V.A.; Presnov, D.E. Thyroglobulin detection by biosensor based on two independent Si NWFETs. *Proc. SPIE-Int. Soc. Opt. Eng.* **2019**, *11022*, 110220Z. https://doi.org/10.1117/12.2522461.
3. Koo, S.M.; Edelstein, M.D.; Li, Q.; Richter, C.A.; Vogel, E.M. Silicon nanowires as enhancement-mode Schottky barrier field-effect transistors. *Nanotechnology* **2005**, *16(9),* 1482. https://dx.doi.org/10.1088/0957-4484/16/9/011
4. Trifonov, A.S.; Presnov, D. E.; Bozhev, I. V.; Evplov, D. A.; Desmaris, V.; and Krupenin, V. A. Non-contact scanning probe technique for electric field measurements based on nanowire field-effect transistor. *Ultramicroscopy* **2017**, *179*, 33–40. https://doi.org/10.1016/j.ultramic.2017.03.030
